# Massive Leiomyomata and Severe Endometriosis Resulting in a Frozen Pelvis in an Asymptomatic Patient

**DOI:** 10.7759/cureus.12097

**Published:** 2020-12-15

**Authors:** Hersh Wazir, Molly S Jain, Enkhmaa Luvsannyam, Michael Rayalu, Charles Alston

**Affiliations:** 1 Medicine, All Saints University College of Medicine, Toronto, CAN; 2 Medicine, Saint James School of Medicine, Park Ridge, USA; 3 Medicine, California Institute of Behavioral Neurosciences and Psychology, Fairfield, USA; 4 Medicine, American University of Integrative Sciences, Atlanta, USA; 5 Obstetrics and Gynecology, Roseland Community Hospital, Chicago, USA

**Keywords:** endometriosis, leiomyoma, fibroids, frozen pelvis, adhesions, ovarian cyst

## Abstract

Leiomyomas, also known as uterine fibroids, are the most common benign uterine tumors in women. The most frequently reported symptoms are uterine bleeding and abdominal and/or pelvic pressure; however, most cases are asymptomatic and may be found incidentally. Endometriosis is a condition where the endometrium proliferates outside of the uterine cavity. Extrauterine endometrial implants are usually found in the ovaries, pelvis, and peritoneum, but can extend anywhere throughout the body. Women with endometriosis may exhibit dysmenorrhea, dyspareunia, dyschezia, and infertility. Inflammation caused by endometriosis may lead to fibrosis, scarring, and adhesions. We report a case of an asymptomatic 36-year-old African-American woman with increasing abdominal girth, consistent with a 28-week gestation, presenting to her obstetrician/gynecologist for her annual exam, who on further investigation is found to have multiple large fibroids, bilateral ovarian cysts, and widespread endometriosis with several adhesions ultimately leading to a frozen pelvis.

## Introduction

Uterine fibroids are the most common gynecologic tumors in women of reproductive age and are seen in more than 77% of all women [1]. Fibroids are benign tumors arising from myometrial smooth muscle cells and can be large, numerous, and found in various locations. Fibroids located in the muscular wall of the uterus are commonly called intramural fibroids and those located within the outer lining or serosa of the uterus are known as subserosal fibroids. Moreover, fibroids inside the interior lining of the uterus are called submucosal fibroids. Symptomatic tumors present with pelvic pain or pressure, abnormal uterine bleeding, anemia, dysmenorrhea, bladder or bowel dysfunction, sexual dysfunction, compression of adjacent pelvic organs, and reproductive disorders such as infertility and recurrent pregnancy loss [2]. The risk factors associated with uterine fibroids are age, early menarche, alcohol, high body mass index (BMI), polycystic ovary syndrome, and race [3,4]. Uterine fibroids are three times more frequently seen in African American women than in Caucasian women [5]. 

Uterine fibroids are diagnosed by transabdominal ultrasound and symptomatic fibroids can be managed medically or surgically. Medical treatment is the preferred option for patients who want to maintain future fertility or patients at high risk for surgery. If symptoms are severe and refractory to medical treatment, myomectomy is the gold standard surgical procedure for women who desire to maintain fertility [2]. Up to 50% of women with fibroids develop severe symptoms requiring surgery [6]. Hysterectomy is reserved for patients who present with severe symptomatic fibroids and do not wish to become pregnant. Uterine fibroids remain to be the most common indication for hysterectomy in the United States with more than 200,000 hysterectomies performed annually [7]. Despite the high prevalence of uterine fibroids and their major complications, there is still not a complete understanding of the pathogenesis of these tumors.

Endometriosis is a complex clinical disease characterized by ectopic implantation of endometrial glands in extrauterine tissues. Endometriosis is seen in 6% to 10% of women of reproductive age and is the most common cause of chronic pelvic pain [8]. Endometriosis is associated with the estrogen-dependent inflammatory process; however, the pathophysiology is not fully understood. The most frequently reported clinical features are chronic pelvic pain, dysmenorrhea, deep dyspareunia, dyschezia, and infertility [9]. Three major forms of endometriosis are peritoneal endometriosis, deep infiltrating endometriosis found in the pouch between the vagina and rectum (pouch of Douglas), and ovarian endometriomas, which present as ovarian cysts filled with blood, commonly known as “chocolate cysts” [10]. 

Initially, the presumptive diagnosis of endometriosis is medically managed with nonsteroidal anti-inflammatory drugs (NSAIDs) and oral contraceptive pills (OCPs), progestins, or gonadotropin-releasing hormone (GnRH) agonists [9]. When symptoms do not improve with medical management, direct laparoscopic visualization is required for definitive diagnosis and resection of implants [9]. Delay in diagnosis is a major problem for the management of endometriosis due to nonspecific symptoms and lack of diagnostic tools. Therefore, the treatment is often not initiated until the disease has progressed at least 8 to 10 years [8].

The chronic inflammation caused by the endometrial glands and stroma found outside the uterine cavity causes remodeling of the surrounding tissues which may lead to fibrosis, scarring, and pelvic adhesions [10]. Extensive adhesions can lead to a frozen pelvis, which is characterized by fibrosis, reduced tissue elasticity, distortion of anatomical structures, severe adhesions, and replacement of pelvic soft tissue by abnormal tissue [11].

In this report, we present a case of a patient with severe adhesions due to widespread endometriosis and large fibroids leading to an asymptomatic frozen pelvis.

## Case presentation

**Figure 1:**
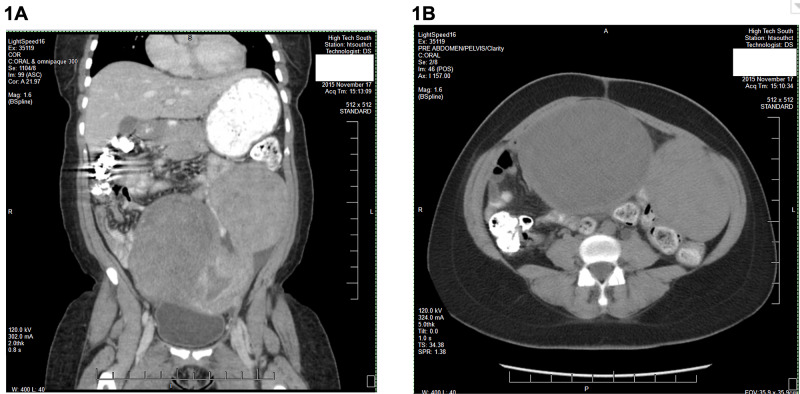
Pelvic CT showing massive fibroid uterus measuring 19 cm in length and 15 cm in width with one large subserosal fibroid projecting superiorly to the right measuring 13 x 12 cm, another projecting superiorly to the left measuring 10 x 8 cm, evidenced by coronal view (A) and again by transverse view (B).

**Figure 2:**
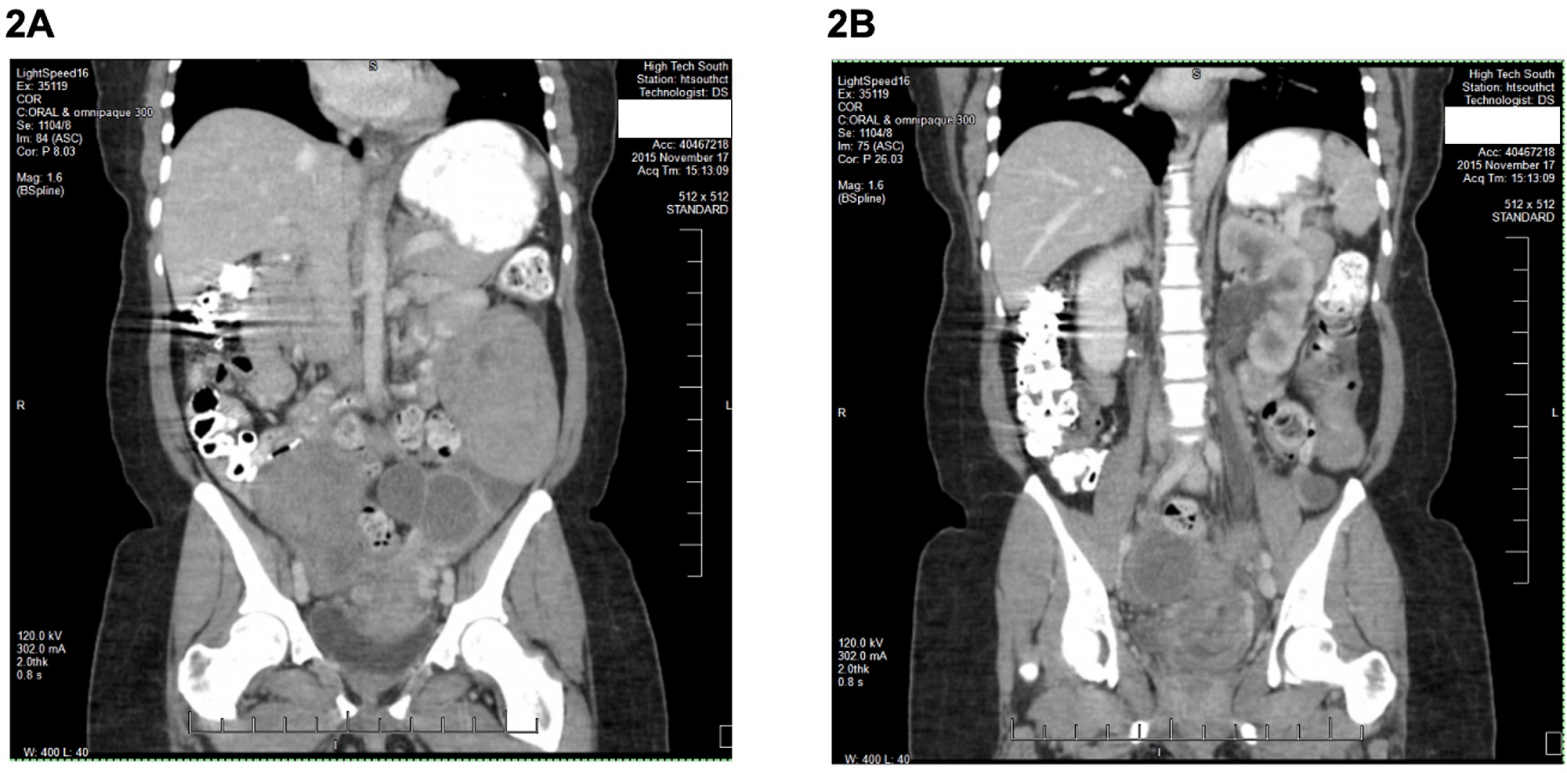
Pelvic CT showing an ovarian multiloculated cyst on the left measuring 8.8 x 6.8 cm (A), and another cystic process on the right measuring 5.1 x 4.1 cm (B).

A nulliparous 36-year-old African-American female, with a past medical history of a recurrent Bartholin cyst and irritable bowel syndrome (IBS), presented to her obstetrician/gynecologist’s (OB/GYN) office for her annual gynecological exam in September 2015. Prior to this, the patient was compliant with her annual appointments which revealed no significant abnormalities. The patient was sexually active with one male partner, with whom she had been trying to get pregnant for two years without success. Both she and her family had noted that her abdomen had increased in girth significantly over the span of six months despite no change in her diet, physical activity, or menstrual cycle, which then prompted her to schedule her annual visit to her OB/GYN office. Her menstrual cycle was regular at 28-day intervals with menses lasting for four days, “like clockwork,” per the patient. The patient stated no irregularities with her menses at this time, denying any menorrhagia, dysmenorrhea, dyspareunia, dyschezia, or dysuria. At the time of her visit, she stated that her abdomen felt firm and her last bowel movement was three days prior, which was thought to be due to her IBS. On abdominal physical exam, firmness was felt across all quadrants, with tenderness in the right lower quadrant upon palpation. The patient described the pain as dull and reported a history of this pain intermittently for two years. She was found to have intra-abdominal and pelvic swelling with findings of a mass and lump at an unspecified site. The remainder of the physical exam revealed no abnormalities. Blood work was drawn at this visit and results were normal aside from an elevated carcinoembryonic antigen (CEA). A computerized tomography (CT) of the abdomen and pelvis was ordered promptly. The results of the CT showed mild hydronephrosis of the right kidney, severe hydronephrosis of the left kidney, a massive fibroid uterus with two dominant subserosal fibroids, and two enlarged ovarian cysts, the left being multiloculated (Figures 1-2).

**Figure 3:**
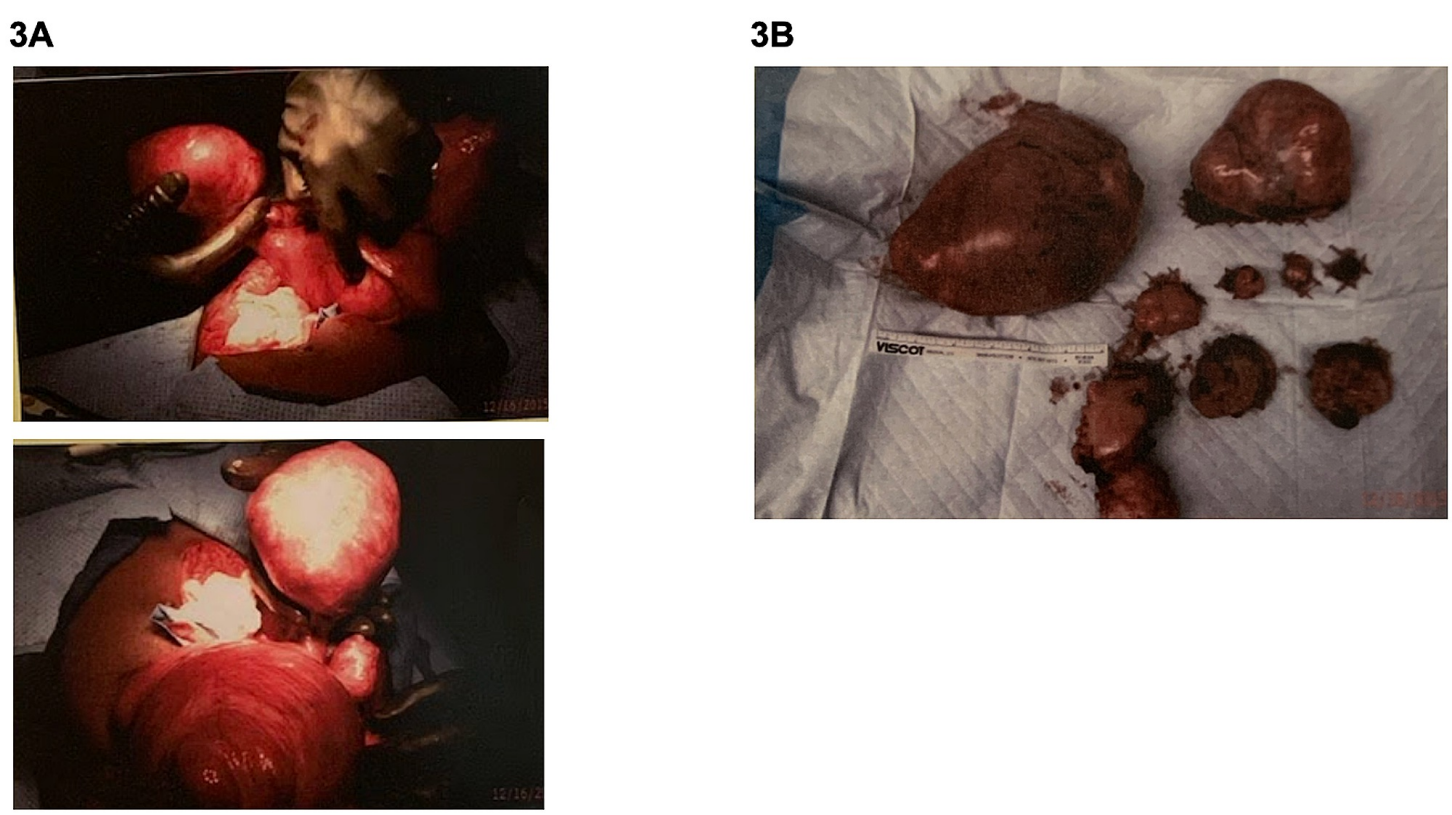
Perioperative images showing a 28-week-gestation sized fibroid uterus during hysterectomy and bilateral salpingo-oophorectomy (A) and massive fibroid uterus with additional subserosal fibroid and bilateral ovarian cysts after resection (B).

The patient was provided with the option to manage the fibroids medically, with Lupron for six months, or surgically via a hysterectomy. After a discussion of these options, their risks, benefits, and their respective prognoses, the patient agreed to a total abdominal hysterectomy as she did not desire to maintain fertility. In December 2015, a total abdominal hysterectomy with bilateral salpingo-oophorectomy with lysis of multiple adhesions of the omentum, intestines, pelvis, ovaries, and rectosigmoid was performed. During the procedure, it was found the uterus was frozen to the pelvic floor, along with several additional fibroids that were not visible on CT (Figure 3). A general surgeon was called in to assist with lysis of numerous adhesions to free the frozen pelvis and colon. The uterus, fallopian tubes, and ovaries were removed. Histopathology revealed chronic cervicitis, proliferative endometrium with endometrial polyps, multiple subserosal leiomyomata, the largest measuring 19 x 16 x 10.5 cm, a patent fallopian tube attached to another subserosal leiomyoma, bilateral ovaries with cysts, one with multiple follicular cysts and the other with a hemorrhagic corpus luteal cyst, and a large leiomyoma weighing 577 g and measuring 13.5 x 11 x 8.5 cm with focal degenerative changes. The patient recovered with no complications from the procedure. Currently, the patient is doing well and takes no medications.

## Discussion

Uterine fibroids are benign tumors in women of childbearing age and the etiology is primarily dependent on hormones, specifically estrogen. The clinical significance of leiomyomas pertains to their size and location in the pelvis. Large fibroids compressing other organs can present with severe symptoms in women causing significant distress to the patient’s quality of life. Surprisingly, our patient did not present with any of the common symptoms and remained asymptomatic despite the later findings of multiple large and heavy fibroids. However, since she had subserosal fibroids, it appears that she remained infertile at least partly due to this reason. Since subserosal fibroids are located outside of the uterus, they can obstruct the pathway to the cervix or fallopian tubes, thereby blocking the journey of sperm towards the fertilized egg for conception [12]. 

Some of the risk factors for the development of uterine leiomyomas are hereditary, others include race, obesity, vitamin D deficiency, and early menarche [13]. Racially, black women are more likely to develop fibroids than women of other races [12]. Moreover, African American women develop fibroids at earlier ages and present with larger tumors with more significant symptoms than Caucasian women [7]. Aside from ultrasound, other imaging modalities include CT, MRI, hysterosonography, hysterosalpingography, and hysteroscopy to locate submucosal leiomyomas and to check the degree of patency of the fallopian tubes [13]. Treatment protocol of uterine fibroids is based on patient symptoms, pregnancy status, and other comorbidities. Current medical treatment for symptomatic fibroids includes NSAIDs, OCPs, high-dose progestins, tranexamic acid, and GnRH agonists [2]. However, medical treatments may cause undesirable side effects, and also cause the tumor to rebound once treatment is stopped [14]. Some minimally invasive procedures such as uterine artery embolization, radiofrequency ablation, laparoscopic myomectomy, hysteroscopic myomectomy, and endometrial ablation can destroy fibroids without their surgical excision [13]. In complicated fibroids, which are larger in size and heavier in weight, surgical procedures are usually required. Due to the size of this patient’s fibroids, the compression of the bilateral ureters, multiple bilateral ovarian cysts, and extensive adhesions, a total abdominal hysterectomy with bilateral salpingo-oophorectomy was performed. This procedure renders an individual infertile but is the permanent solution to complicated fibroids as seen in this case.

Furthermore, while the hysterectomy was being performed, there was an unexpected finding of severe endometriosis of the pelvis, ovary, uterus, and rectosigmoid colon with multiple adhesions of the omentum. The patient was peri-operatively diagnosed with a severe frozen pelvis as a result of the aforementioned complications. The endometriotic implants could be grown anywhere from the ovaries and bowel to any tissues lining the pelvis. The pathophysiology behind endometriosis is quite complex, and there are still many theories. The retrograde menstruation theory states that the endometrial cells flow backward into the pelvic cavity through the fallopian tubes during menses, implant onto various abdominal organs and peritoneum, grow, and cause chronic inflammation causing adhesions [9]. The severity of the disease is associated with the amount of menstrual flow, estrogen dependence, progesterone resistance, inflammation, and genetic and environmental factors [9].

Diagnosing endometriosis involves taking a detailed history, performing pelvic exams, implementing transvaginal/abdominal ultrasound, and finally, carrying out laparoscopy [15]. Diagnosis and medical management of endometriosis involve enacting a similar approach to that of leiomyomata. There are four stages of endometriosis: minimal, mild, moderate, and severe. The severe stage of endometriosis is apparent when the implants are quite deep within the pelvic cavity and ovaries. The culmination of the severe stage may result in a frozen pelvis; this occurs when the pelvic organs, such as the rectum, bladder, ureters, large and small bowels, and/or ovaries become densely adhered to one another via adhesions [16]. The organs that are densely adherent may have nerves that develop within the adhesions, leading to neuropathic pain which may be masked by the pain of endometriosis [15]. Frozen pelvis requires prompt surgery to alleviate the aforementioned adhesions and symptoms.

The highlight of this case is the asymptomatic presentation of the patient even though she had multiple life-threatening gynecological disorders including multiple large fibroids along with severe endometriosis leading to a frozen pelvis. The patient was not taking any medications and felt nothing abnormal in spite of having such severe pelvic conditions. The patient faced infertility which could be attributed to the combined effect of both the fibroids and endometriosis as well as the adhesions. This case is rare, as it points to the infrequent presentation of multiple common gynecological conditions. It also highlights the importance of regular physical exams and follow-ups to prevent severe complications such as infertility, bowel obstructions, kidney injury, and even hemorrhage.

## Conclusions

Our understanding of uterine leiomyomas and endometriosis often involves symptomatic review, as well as our knowledge that patients differ in severity. It is thus a rare case where a patient is severely affected but oddly asymptomatic. The woman in this case presented with moderate distention while CT showed multiple, massive fibroids that would normally present with severe symptoms. Additionally, when clinically appraised, the patient was found to have further pelvic adhesions and multiple foci of endometriosis throughout her pelvic organs and on her bowel. The question could be asked, which gynecologic pathology arose first? A more important question is asking, how can we adapt to what amounts to very individual experiences in patient symptomatology? This case highlights the importance of doing so and how paramount it is for clinicians to pay attention to early diagnosis regardless of type or severity of symptoms, especially in patients such as this with multiple risk factors.

## References

[1] Cramer SF, Patel A (1990). The frequency of uterine leiomyomas. Am J Clin Pathol.

[2] Segars JH, Parrott EC, Nagel JD (2014). Proceedings from the Third National Institutes of Health International Congress on Advances in Uterine Leiomyoma Research: comprehensive review, conference summary and future recommendations. Hum Reprod Update.

[3] Wise LA, Palmer JR, Harlow BL, Spiegelman D, Stewart EA, Adams-Campbell LL, Rosenberg L (2004). Risk of uterine leiomyomata in relation to tobacco, alcohol and caffeine consumption in the Black Women's Health Study. Hum Reprod.

[4] Wise LA, Palmer JR, Spiegelman D (2005). Influence of body size and body fat distribution on risk of uterine leiomyomata in U.S. black women. Epidemiology.

[5] Marshall LM, Spiegelman D, Barbieri RL (1997). Variation in the incidence of uterine leiomyoma among premenopausal women by age and race. Obstet Gynecol.

[6] Lethaby A, Vollenhoven B, Sowter MC (2001). Pre-operative GnRH analogue therapy before hysterectomy or myomectomy for uterine fibroids. Cochrane Database Syst Rev.

[7] Moravek MB, Yin P, Ono M (2015). Ovarian steroids, stem cells and uterine leiomyoma: therapeutic implications. Hum Reprod Update.

[8] Gemmell LC, Webster KE, Kirtley S, Vincent K, Zondervan KT, Becker CM (2017). The management of menopause in women with a history of endometriosis: a systematic review. Hum Reprod Update.

[9] Vercellini P, Viganò P, Somigliana E, Fedele L (2014). Endometriosis: pathogenesis and treatment. Nat Rev Endocrinol.

[10] Bulun SE, Yilmaz BD, Sison C (2019). Endometriosis. Endocr Rev.

[11] Keckstein J, Becker CM, Canis M (2020). Recommendations for the surgical treatment of endometriosis. Part 2: deep endometriosis. Hum Reprod Open.

[12] Williams ARW (2017). Uterine fibroids - what's new?. F1000Res.

[13] Barjon K, Mikhail LN (2020). Uterine Leiomyomata. https://www.ncbi.nlm.nih.gov/books/NBK546680/.

[14] Walker CL, Stewart EA (2005). Uterine fibroids: the elephant in the room. Science.

[15] Parasar P, Ozcan P, Terry KL (2017). Endometriosis: epidemiology, diagnosis and clinical management. Curr Obstet Gynecol Rep.

[16] Alimi Y, Iwanaga J, Loukas M, Tubbs RS (2018). The clinical anatomy of endometriosis: a review. Cureus.

